# P81 Interim analytical validation of rapid microcapillary antimicrobial susceptibility testing for Gram-positive pathogens

**DOI:** 10.1093/jacamr/dlaf230.088

**Published:** 2025-12-04

**Authors:** Julie Hart, Sarah Helen Needs, Oliver Hancox, Alexander Edwards

**Affiliations:** University of Reading, Reading, UK; Astratus Limited, Reading, UK; University of Reading, Reading, UK; Astratus Limited, Reading, UK; University of Reading, Reading, UK; Astratus Limited, Reading, UK; University of Reading, Reading, UK; Astratus Limited, Reading, UK; University of Southampton, Southampton, UK

## Abstract

**Background:**

Standard culture methods for antimicrobial susceptibility testing (AST) are manual and laborious. Fast, higher throughput, digital and automated AST is urgently required for veterinary and human health. Astratus Limited was spun out from the University of Reading to commercialize our novel, affordable microcapillary AST platform. The modular instrument design allows medium to ultra-high-throughput rapid AST options for both direct-from-urine and isolate testing. We previously presented the analytical performance of direct-from-urine microcapillary AST with >450 human urine samples showing 96% sensitivity (95% CI: 92%–99%) and 93% specificity (95% CI: 89%–96%) for detecting microbial growth in urine with >105 cfu/mL. Direct-from-urine microcapillary AST showed a concordance with broth microdilution of 557/572 bacteria/antibiotic combinations (97.38%) for urine samples with a single Gram-negative bacteria. The mean time to AST result was 5.7 h from 85 positive urine samples. Analytical performance data for Gram-positive bacteria is equally critical. Our objective was to analytically validate microcapillary AST with Gram-positive bacterial isolates.

**Methods:**

Analytical validation of microcapillary AST was conducted with a total of 425 antibiotic/Gram-positive isolates: *Staphylococcus* and *Enterococcus* isolates from human urine samples (Hampshire Hospitals, UK); and *Staphylococcus* isolates from canine urine, pus and wound samples (Batt Laboratories, UK). Human isolates were tested side-by-side, microcapillary AST was compared to the in-house reference broth microdilution method (BMD). Canine isolate microcapillary AST results were compared to Batt Laboratories reported results. In this study 18 antibiotics were tested simultaneously using microcapillary AST for continuous growth measurement: Ampicillin; Amoxicillin clavulanic acid; Nitrofurantoin; Clindamycin; Doxycycline; Gentamicin; Oxacillin; Enrofloxacin; Penicillin G; Erythromycin; Cephalexin; Cefoxitin.

**Results:**

High categorical agreement was observed indicating excellent accuracy for both human (128/135; 95%) and canine (265/290; 91%) Gram-positive isolates. Closer agreement was seen for human isolates tested with microcapillary AST compared to in-house BMD than for canine isolates tested with microcapillary AST compared to laboratory-reported results. However, overall accuracy remained high for canine isolates. The difference in accuracy was found to be related to an increased number of discordant results for certain antibiotics within the canine isolate dataset. For seven of the antibiotics tested, accuracy exceeded 90%. Four antibiotics showed lower agreement (ampicillin, penicillin G, erythromycin and cephalexin).

**Conclusions:**

Detailed analysis of discordant results is planned alongside side-by-side comparison of canine isolates tested with microcapillary AST compared to in-house BMD to remove the laboratory-to-laboratory variation. Further optimization of the system may be required to ensure that the accuracy of the AST results for the 4 antibiotics matches the high accuracy of the AST results of the other antibiotics so that we can ensure that we provide faster, accurate antimicrobial susceptibility testing results to enable more targeted antimicrobial prescribing. Our aim is to replace manual, labour intensive methods with a single, simple-to-use, higher throughput platform unlocking productivity and relieving laboratory workforce pressures across human and veterinary medicine.

